# Clinical performance of AED shock advisory system with integrated Analyze Whilst Compressing algorithm for analysis of the ECG rhythm during out-of-hospital cardiopulmonary resuscitation: A secondary analysis of the DEFI 2022 study

**DOI:** 10.1016/j.resplu.2024.100740

**Published:** 2024-08-05

**Authors:** Jean-Philippe Didon, Irena Jekova, Benoît Frattini, Sarah Ménétré, Clément Derkenne, Vivien Hong Tuan Ha, Daniel Jost, Vessela Krasteva

**Affiliations:** a Schiller Médical SAS, 4 rue L. Pasteur, 67160 Wissembourg, France; b Institute of Biophysics and Biomedical Engineering, Bulgarian Academy of Sciences, Acad. G. Bonchev Str. Bl 105, 1113 Sofia, Bulgaria; c Paris Fire Brigade, 1 place Jules Renard, 75017 Paris, France

**Keywords:** Automated external defibrillator (AED), Cardiopulmonary resuscitation (CPR), Cardiac arrest, Chest compression, Ventricular fibrillation, Algorithm, CPR artifact, ECG analysis

## Abstract

**Objective:**

This study involving automated external defibrillators (AEDs) in early treatment of refibrillation aims to evaluate the performance of a new shock advisory system (SAS) during chest compressions (CC) in out-of-hospital cardiac arrest (OHCA) patients.

**Methods:**

This work focuses on AED SAS performance as a secondary outcome of DEFI 2022 clinical prospective study, which included first-analysis shockable OHCA patients. SAS employs the Analyze Whilst Compressing (AWC) algorithm to interact with both cardiopulmonary resuscitation (CPR) and shock advice by conditional operation of two-stage ECG analysis in presence or absence of chest compressions. AWC is triggered by the first-shock recommendation. Then, after 1 min of CPR, ECG analysis during CC decides between two treatment scenarios. For patients with refibrillation, CPR is paused for immediate confirmation analysis and shock advice. For patients with non-shockable rhythms, CPR is continued for 2 min until standard analysis.

**Results:**

Clinical data from 285 OHCA patients with shock recommendation at the first-analysis by AEDs (DEFIGARD TOUCH7, Schiller Médical) consisted of 576 standard analyses, 2011 analyses during CC, 577 confirmation analyses in absence of CC. Global AED SAS performance meets the standard recommendations for arrhythmia analysis sensitivity (94.9%) and specificity (>99.3%). AWC provided innovative treatment of shockable rhythms by stopping CPR earlier than 2 min in most ventricular fibrillations (92.9%), while most non-shockable patients (86.5–95.2%) benefitted from continuous CPR for at least 2 min.

**Conclusion:**

This study provides positive evidence for routine use of AEDs with AWC-integrated algorithm for ECG analysis during CPR by first-responders in early OHCA treatment.

**Clinical Trial Registration:** Registration number: NCT04691089, trial register: ClinicalTrials.gov.

## Introduction

Survival from out-of-hospital cardiac arrest (OHCA) is low despite some improvements over the last decades.^1^ OHCA patients are treated with a combination of chest compressions (CC) and ventilations for 2 min (cardiopulmonary resuscitation, CPR) and defibrillation shocks according to the Basic Life Support (BLS) algorithm.^2^, ^3^ Post-shock refibrillation is very common, with incidence over 50% after the initial shock terminating ventricular fibrillation (VF).^4^, ^5^ A recent study provides evidence that 82% of refibrillations occurred within the first minute post-shock.^6^, ^7^ Rhythm analysis during the CPR phase may allow reducing the time the patient spends in VF, but this should not be at the expense of a substantial increase in the hands-off time due to systematic interruptions of CPR earlier than 2 min. Therefore, the shock advisory system (SAS) in automated external defibrillators (AEDs) needs to incorporate specific algorithms that robustly analyse the electrocardiogram (ECG) during CC artifacts to avoid unnecessary CPR stopping.

The development of SAS algorithms during CPR is an extensively studied topic, lately managed by machine learning and deep learning algorithms,^8^, ^9^, ^10^, ^11^, ^12^, ^13^, ^14^, ^15^ still without evidence of implementation in AEDs. Several commercial AED SAS algorithms provide different schemes for triggering two-stage rhythm analysis during CPR, applying the first stage during uninterrupted CC (analysis duration 11–30 s), eventually followed by a second confirmatory stage on clean-ECG (5–9 s).^16^, ^17^, ^18^, ^19^ The main difference between the protocols covers SAS decisions during CPR that activate the confirmation stage after detection of shockable,^16^, ^17^ non-shockable^17^ or undecided rhythms.^18^, ^19^ A flow-chart of each two-step SAS algorithm can be found in Didon et al.^16^ Three studies^16^, ^18^, ^19^ conclude about the potential of respective schemes for improving the CPR quality by shortening pre-shock pauses in most VF patients and not interrupting CC in most non-VF patients. The above conclusions are based on OHCA data analysed in two *in silico* studies^16^, ^18^ and one clinical study with AED embedded algorithm.^19^

Quality of patient care is assessed by a registered clinical study (DEFI 2022) using a new defibrillation algorithm.^20^ This secondary analysis of the DEFI 2022 study is focused on detailed performance of SAS, namely Analyze Whilst Compressing (AWC) algorithm, conditionally triggered in presence and absence of CC. This performance is compared with another clinical SAS study.^19^

## Materials and methods

### Patient population and study design

This work is a secondary analysis of DEFI 2022 (ClinicalTrials: NCT04691089), which compared CPR quality of professional rescuers after implementation of a defibrillation algorithm for early treatment of refibrillation in 2022 versus a historical cohort from 2017. DEFI 2022 study was managed by the Emergency Medical Service of the Paris Fire Brigade (BSPP) in Greater Paris area. The BSPP professional first-responders participated in the BLS of early OHCA treatment by using 389 commercial AEDs (DEFIGARD Touch7 DGT7, Schiller Médical) equipped with a new AWC algorithm interacting with both CPR and shock advice. The resuscitation protocol was provided with 30:2 compression-to-ventilation ratio and a metronome guided compression rate of 110 min^−1^. This study was approved by the French Institutional Review Board, number 20.01291.202018. Patients or their families were informed about the study’s terms and patients’ data with absence of consent were excluded from the study analysis.

In this study, the 2022 cohort (from 01/01/2021 to 31/01/2022) of 285 consecutive patients, enrolled in the interventional arm of DEFI 2022, included non-traumatic OHCA in patients over 12 years of age, treated by a BLS team, with a shockable rhythm identified by AED at the first rhythm analysis. The exclusion criteria were: AED used in pediatric mode; patients treated with an on-site AED; patients with a pacemaker; withdrawn consent of patients or their families to use the data; interventions with unreadable electrocardiographic (ECG) or transthoracic impedance data.

Given the practice that adult advanced life support teams arrive usually more than 10 min after BLS teams, the observation window was limited to 10 min or less if return of spontaneous circulation (ROSC) was identified or AED was disconnected.

### AWC algorithm

**Fig. 1:**
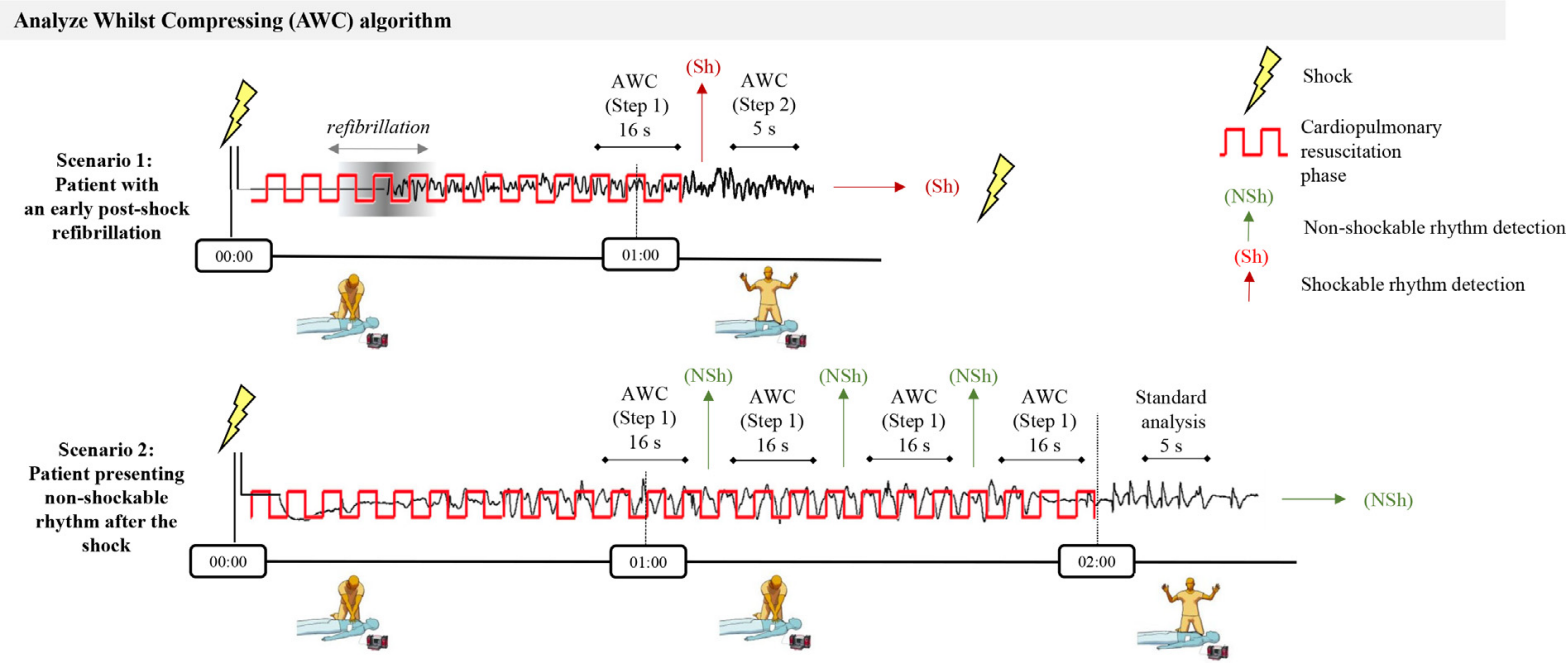
Scheme of the AWC algorithm embedded in AED shock advisory system, managing shock delivery by sequential activation of different ECG rhythm analysis stages. The first shock advisory result is taken after 1 min of CPR-phase by AWC(Step1), deciding on two treatment scenarios: Scenario 1 in a patient with an early post-shock refibrillation: CPR is stopped for immediate confirmation analysis by the AWC (Step2), which decides to administer early shock. Scenario 2 in a patient with a non-shockable rhythm: After the conventional 2-minute CPR-period, the Standard analysis conducted in absence of CC decides to resume CPR.

In this study, the first rhythm analysis was performed by an AED in absence of CC, further referred as “Standard analysis”. Regardless of whether a shock was administered, CPR was immediately resumed for a maximum of 2 min. The duration of the CPR-phase was managed by the patent protected Analyze Whilst Compressing (AWC) algorithm for AEDs^21^, ^22^ designed with the presumption of early treatment of refibrillation, without impairing the chest compression fraction for patients not presenting with shockable rhythms. The AWC algorithm (Fig. 1) includes several rhythm analysis stages, sequentially triggered in presence and absence of CC:

· AWC(Step1) is a rhythm analysis stage in presence of CC (analysis duration 16 s). Its shock advisory result is considered when given at least 1 min after the beginning of the CPR-phase. This time delay is consistent with clinical evidence that 82% of refibrillations occur within the first post-shock minute.^6^, ^7^ As long as a non-shockable rhythm is repeatedly detected, AWC(Step1) is activated regularly until the end of the 2-minute CPR-phase. The rhythm analysis technology during CC is based on a single ECG lead signal processing.^23^
· AWC(Step2) is a shock confirmation analysis (CoA) in absence of CC (analysis duration 5 s), activated when AWC(Step1) detects shockable rhythm. CoA is triggered with minimal delay after chest compressions are stopped.^24^ AWC(Step2) can either confirm the need for shock delivery or advise resuming CPR.
· Standard analysis (analysis duration 5 s) is activated after the 2-minute CPR-phase if AWC(Step1) repeatedly detects non-shockable rhythm. It can either recommend a shock or confirm the non-shockable rhythm that requires immediate CPR resumption. The ECG processing algorithm of the Standard analysis is validated in previous studies.^19^, ^25^, ^26^

**Fig. 2:**
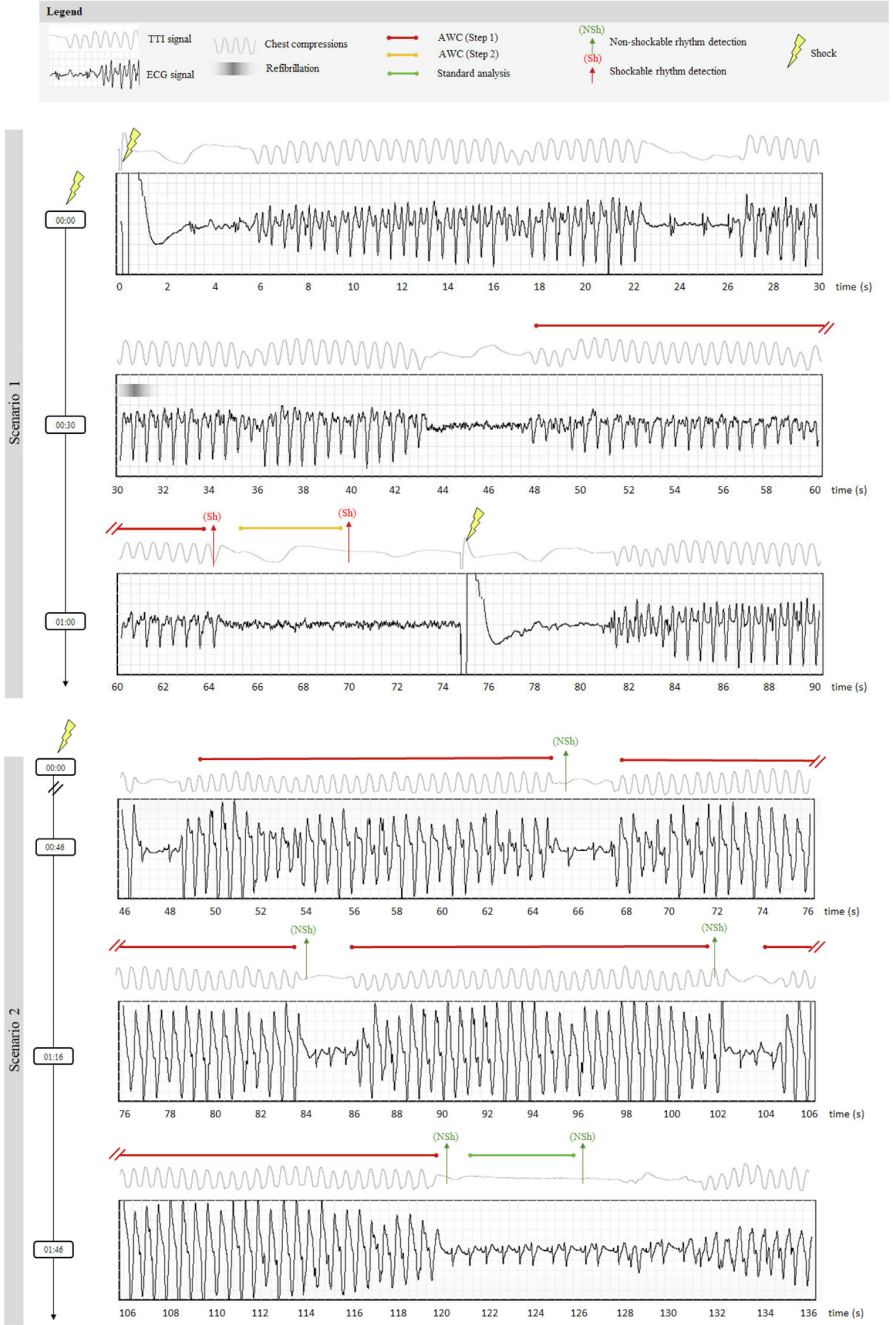
Recorded CPR phases of two interventions, following the basic treatment schemes disclosed in Fig. 1. In both, the CPR phase started immediately after the shock, denoted at time (00:00) on the vertical time-axis. Each scenario is illustrated with sequential signal traces (ECG: Electrocardiogram, TTI: Transthoracic impedance) and timing of different AED events. Scenario 1 was recorded in a patient with early post-shock refibrillation (observed about 30 s after the shock, during CC). The analysis of AWC (Step1) was activated at 48–64 s and the detected shockable rhythm stopped the CPR phase. Then the shock was confirmed by AWC (Step2) at 70 s and early shock was delivered at 75 s. The new CPR phase was then resumed. Scenario 2 was recorded in a patient with non-shockable rhythm after the shock, which was detected by four analyses of AWC (Step1), sequentially triggered during CC at 49–65 s, 68–84 s, 86–102 s, 104–120 s after the shock. The CPR phase was conditionally stopped at 2 min for the regular rhythm check by the Standard analysis (121–126 s). The non-shockable rhythm was confirmed, and the next CPR phase was resumed at 131 s.

The effect of AWC on the CPR-phase duration is illustrated in Fig. 1. In a scenario with early post-shock refibrillation (Scenario 1), the CPR-phase can be shortened to 1 min if AWC(Step1) has detected shockable rhythm and thus an early shock can be delivered. Otherwise, the CPR-phase can be extended to 2 min (Scenario 2), thus preserving chest compression fraction for patients presenting non-shockable rhythms. Derived from OHCA study interventions, the clinical application of the AWC algorithm is illustrated in Fig. 2 with two examples of recorded analysis and CPR phases. Those are corresponding to the basic treatment schemes of Scenario1 and Scenario2 in Fig. 1.

### Data collection and rhythm annotation

The electronic records stored in AEDs contain ECG and transthoracic impedance signals acquired through anterolateral defibrillation pads, sampled at 500 Hz. Different events detected by the real-time processes in AEDs were also recorded, including CC-start and CC-stop times, AWC rhythm analysis periods and respective shock advisory decisions throughout the intervention. The site of investigation assured the anonymization of transmitted electronic and patient data (epidemiological, clinical, survival).

Three emergency physicians with experience in cardiac arrest cardiology from BSPP visually revised the unfiltered ECG and transthoracic impedance traces in an annotation window, which matches the AED analysis periods in presence and absence of CC. Additionally, the annotations during CC might use evidence of the rhythm observed in insufflation pauses. A consensus decision was asked in cases of rhythm annotation disagreement.

The annotations followed the American Heart Association (AHA) rhythm classification scheme,^27^ including five rhythm categories with performance goal recommendation for detection of shockable and non-shockable rhythms by AEDs in absence of artifacts: (1) VF with amplitude >200 µV; (2) rapid ventricular tachycardia (VT) with heart-rate ≥150 min^−1^; (3) normal sinus rhythm (NSR); (4) other organized non-shockable rhythms (ONR), including supraventricular tachycardia, sinus bradycardia, atrial fibrillation/flutter, heart blocks, idioventricular rhythms and premature ventricular contractions, heart-rate >30 min^−1^; (5) asystole (ASYS) with peak-to-peak amplitude <100 µV, lasting >4 s. Intermediate rhythms were annotated as: (1) fine-VF with amplitude 100–200 µV; (2) slow-VT with heart-rate <150 min^−1^; (3) rhythm transitions within the analysis window (during AWC(Step1) or AWC(Step2)) with noticed rhythm changes from non-shockable to shockable or vice versa. When a consensus decision could not be taken, the rhythm was annotated as undefined and excluded.

### Outcomes and statistical analysis

The first outcome was the global AED SAS performance during CPR. Additionally, the performance of each analysis stage was disclosed (Standard analysis, AWC(Step1), AWC(Step2)). Explanations and formulas are presented in Table S3.

The shock-advisory performance was estimated by the method of Zhou,^28^, ^29^ which is applied for clusters of multiple analyses per patient. It was statistically evaluated in terms of sensitivity for shockable rhythms (VF, rapid-VT), specificity for non-shockable rhythms (NSR, ONR, ASYS) and the one-sided 90% lower confidence limit (LCL90). The method of Zhou was applied by considering multiple CPR episodes from a single AED intervention. Such data augmentation is a common practice given that the CPR process depends on non-stationary factors, such as the patient’s treatment (shock delivery, drug injection, etc.), CC delivery (fatigue of the rescuer, swap of rescuers, etc.) and patient’s cardiac response (amplitude and frequency ECG variations over time, refibrillation, ROSC, etc.).

Confirmation rate was estimated as the percentage of cases needing CoA among all AWC(Step1) analyses during CC. The rate of patients who received ill-advised CoA (shock advice by AWC(Step1) in presence of a non-shockable rhythm) was also measured as a second outcome, to verify the safety of AWC in patients presenting non-shockable rhythms during CC. As a complement, the hands-off time of each ill-advised CoA was quantified.

The descriptive statistics report discrete and continuous variables as percentages and median values [interquartile ranges, IQR], respectively.

## Results

### OHCA interventions

This study enrolled 285 consecutive OHCA patients (81% men, aged 60 [51–72] years, median [IQR]). All patients had a shock recommendation at the first analysis. Patient baseline characteristics are given in Table S1.

**Fig. 3:**
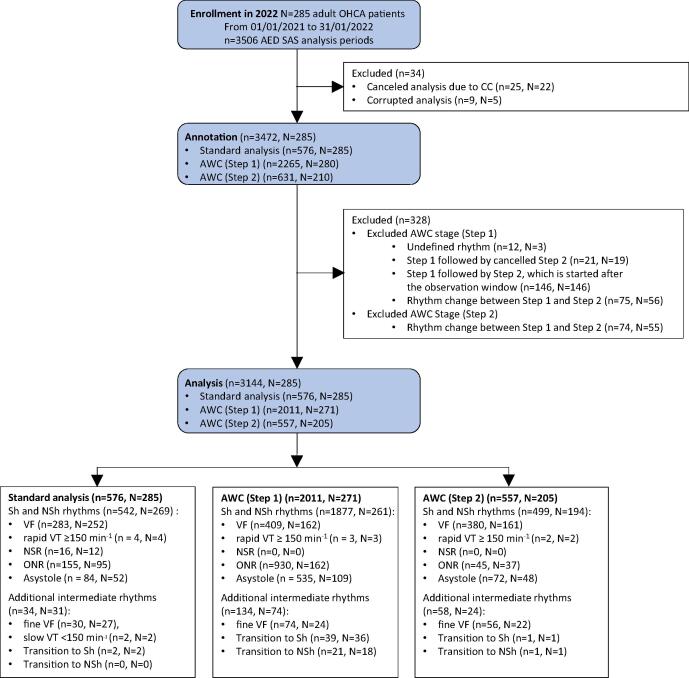
Flowchart of patient enrollment, annotation and analysis. AED: Automated external defibrillator; SAS: Shock advisory system; AWC: Analyze Whilst Compressing algorithm; Sh: Shockable; NSh: Non-shockable; VF: Ventricular fibrillation; VT: Ventricular tachycardia; NSR: Normal sinus rhythm; ONR: Other non-shockable rhythm.

Analysis of all 285 interventions identified a total of 3506 AED analysis periods with recorded SAS decisions. Among those, 362 cases were excluded (Fig. 3) and the outcomes were analysed using 3144 SAS decisions (576 Standard analysis, 2011 AWC(Step1), 557 AWC(Step2)).

### Shock advisory performance

The results for the first outcome are shown in Table 1A. The global AED SAS performance during CPR was computed from all decisions of Standard analysis and AWC(Step1 + Step2). The sensitivity of the AED was 94.9% for shockable rhythm and specificity is 100%, 99.7% and 99.3%, respectively for normal sinus rhythm, asystole and other non-shockable rhythms.

**Table 1:** Rhythm detection performance of AED shock advisory system during CPR in this clinical study. AWC: Analyze Whilst Compressing algorithm; Se: Sensitivity; Sp: Specificity; LCL90: one-sided 90% lower confidence limit; VF: Ventricular fibrillation; NSR: Normal sinus rhythm; ONR: Other non-shockable rhythm; ASYS: Asystole.

A. Global shock advisory system performance: Standard analysis + AWC (Step 1 + Step 2).							
Rhythm	Patients	Analyses	Shock advised	No shock advised	Performance (Se, Sp)	LCL90	Performance Goal^27^, ^32^
VF	260	692	657	35	94.9%	93.7%	>90%
NSR	12	16	0	16	100%	100%	>99%
ONR	167	1085	8	1077	99.3%	99.0%	>95%
ASYS	112	619	2	617	99.7%	99.4%	>95%

B. Detailed performance of each ECG analysis stage.						
Algorithm/Rhythm	Patients	Analyses	Shock advised	No shock advised	Performance (Se, Sp)	LCL90
Analysis during clean ECG: Standard analysis						
VF	252	283	283	0	100%	100%
NSR	12	16	0	16	100%	100%
ONR	95	155	5	150	96.8%	93.6%
ASYS	52	84	2	82	97.6%	92.8%
Regular analysis in presence of chest compressions: AWC (Step 1)						
VF	162	409	380	29	92.9%	90.3%
NSR	0	0	−	−	NA	NA
ONR	162	930	45	885	95.2%	94.4%
ASYS	109	535	72	463	86.5%	84.2%
Confirmation analysis in absence of chest compressions: AWC (Step 2)						
VF	161	380	374	6	98.4%	97.5%
NSR	0	0	−	−	NA	NA
ONR	37	45	3	42	93.3%	78.8%
ASYS	48	72	0	72	100%	100%
Analyses sequence: AWC (Step 1 + Step 2)						
VF	162	409	374	35	91.4%	88.7%
NSR	0	0	−	−	NA	NA
ONR	162	930	3	927	99.7%	99.5%
ASYS	109	535	0	535	100%	100%

SAS performance on clean-ECG signals was 99.1% (657/663) for VF sensitivity, 96% (192/200) for ONR specificity and 98.7% (154/156) for ASYS specificity, where numbers are derived from the summary performance of Standard analysis and AWC (Step2), (Table 1B).

SAS performance on CC-corrupted-ECG signals was 92.9% for VF sensitivity, 95.2% for ONR specificity and 86.5% for ASYS specificity (Table 1B). Combining information from Table 1B, underscores that only 8% (45 ONR + 72 ASYS) of non-shockable cases (930 ONR + 535 ASYS) required analysis confirmation by AWC(Step2). Thus, shock was rejected for 100% of ASYS and 99.7% of ONR, considering the sequential analysis of AWC(Step1 + Step2).

Table 2 compares the SAS performance found in this study versus another integrated AED algorithm (cprINSIGHT, LIFEPAK CR2, Stryker).^19^

**Table 2:** Rhythm detection performance of AED shock advisory systems during CPR in clinical studies: Comparison of AWC vs. cprINSIGHT algorithms (results are computed from data in original article).

	This study	De Graaf et al. (2021)^19^
AED used for data collection	DEFIGARD Touch7 DGT7 (SCHILLER Médical)	Lifepack CR2 (STRYKER)
Name of the algorithm (study type)	AWC (clinical)	cprINSIGHT (clinical)
Strategy of the embedded algorithm	To improve the treatment of refibrillation (administer an early shock in shockable patients)	To provide continuous CPR at 2-minutes for non-shockable patients
Number of patients	285	365
Number of analysis strips (validation dataset)	2412	726*
Duration of analysis during CC (Step 1)	16 s	30 s
Duration of analysis during reconfirmation (Step 2)	5 s	10 s
Confirmation rate**	**27.7%** (557/2011)	**29.6%** (215/726)
Performance of analysis during clean ECG: Standard analysis		
VF (Sensitivity)	**100%** (283/283)	
NSR (Specificity)	**100%** (16/16)	NA
ONR (Specificity)	**96.8%** (150/155)	
ASYS (Specificity)	**97.6%** (82/84)	
Performance of regular analysis in presence of chest compressions (Step 1)		
VF (Sensitivity)	**92.9%** (380/409)	**81.8%** (90/110)
NSR (Specificity)	**NA** (0/0)	NA (0/0)
ONR (Specificity)	**95.2%** (885/930)	**78.2%** (169/216)
ASYS (Specificity)	**86.5%** (463/535)	**60.3%** (240/398)
Performance of reconfirmation analysis in absence of chest compressions (Step 2)		
VF (Sensitivity)	**98.4%** (374/380)	**100%** (15/15)
NSR (Specificity)	**NA** (0/0)	NA (0/0)
ONR (Specificity)	**93.3%** (42/45)	**97.8%** (46/47)
ASYS (Specificity)	**100%** (72/72)	**97.4%** (148/152)
Performance of analyses sequence (Step 1 + Step 2)		
VF (Sensitivity)	**91.4%** (374/409)	**95.4%** (105/110)
NSR (Specificity)	**NA** (0/0)	NA (0/0)
ONR (Specificity)	**99.7%** (927/930)	**99.5%** (215/216)
ASYS (Specificity)	**100%** (535/535)	**97.5%** (388/398)
All rhythms (Accuracy)***	**98.0%** (1836/1874)	**97.8%** (708/724)
Global shock advisory system performance: Standard analysis + AWC (Step 1 + Step 2)		
VF (Sensitivity)	**94.9%** (657/692)	
NSR (Specificity)	**100%** (16/16)	NA
ONR (Specificity)	**99.3%** (1077/1085)	
ASYS (Specificity)	**99.7%** (617/619)	

NA: Not applicable or not reported; VF: Ventricular fibrillation; NSR: Normal sinus rhythm; ONR: Other non-shockable rhythm; ASYS: Asystole.

* The number of strips represents all cases analyzed in Step 1 plus two pulseless ventricular tachycardias for which performance was not disclosed.

** Confirmation rate is estimated as the percentage of cases needing a confirmation analysis (Step 2) among all analyses during CC (Step 1).

*** Accuracy related to performance of analyses sequence (Step 1 + Step 2) is the sum of true positives and true negatives divided by the total number of analysis strips.

### AWC safety

**Fig. 4:**
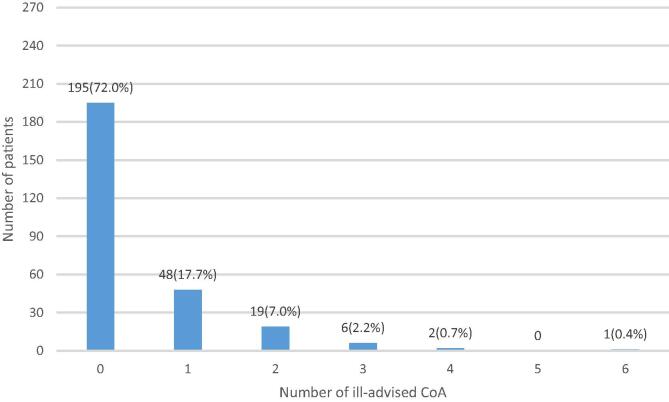
AWC algorithm safety: Histogram of the analysed population of 271 patients in bins for the number of ill-advised CoA by the AWC (Step 1) during chest compressions. The dominant bin at label “0” represents the correctly advised 195 patients (72%). At least one ill-advised CoA was found in 76 patients (28%), who presented non-shockable rhythms in presence of chest compressions: 48 (17.7%) had only one ill-advised CoA, 19 (7%) had two ill-advised CoA, 6 (2.2%) had three ill-advised CoA, 2 (0.7%) had four ill-advised CoA, and 1 (0.4%) had six ill-advised CoA. AWC: Analyze Whilst Compressing algorithm; CoA: shock Confirmation Analysis.

According to Fig. 3, among 285 enrolled patients, only 271 underwent AWC(Step1) and 205 underwent AWC(Step2). At least one ill-advised CoA was found in 76 patients (28%), who presented non-shockable rhythms during CC (Step1), (Fig. 4). The remaining 195 patients (72%) were correctly advised. The global amount of ill-advised CoA was 118, which leads to an ill-advised confirmation rate of 5.9% (118/2011) and a median hands-off time of 10 s [IQR: 10–12 s]. The rhythm distribution during CC implied with ill-advised CoA was: 72 (61%) asystole, 45 (38%) ONR, 1 (0.85%) transition from shockable to non-shockable.

## Discussion

A relationship between minimized shock time and shock duration has been shown since 2007,^30^ calling into question the current guideline recommendations of 2 min of CPR between defibrillation attempts. Recently, Awad et al.^31^ performed a study that aimed to investigate the relationship between the duration of intra-arrest VF/VT and ROSC in OHCA patients. The study found that longer durations of VF/VT were significantly associated with a lower chance of achieving ROSC. In conclusion, these findings suggest a need to reevaluate the current OHCA resuscitation guidelines, recommending a 2-minute interval for rhythm checks and shock delivery.

AED protocols for analyzing heart rhythm status during CPR generally follow two strategies. Conventional AEDs assess the ECG signal every 2 min, pausing CPR for analysis. This strategy is advantageous in avoiding interruption of CPR, if the patient's rhythm does not require a shock.^19^ However, when the patient presents early refibrillation during CPR, the necessary shock is delayed. Conversely, AEDs equipped with the AWC algorithm continuously analyze the ECG rhythm during CPR. In our study, this resulted in an earlier shock for most VF patients (91.4%), while most non-shockable patients (86.5% for ASYS, 95.2% for ONR) benefitted from continuous CPR for at least 2 min. Due to the high sensitivity of the AWC algorithm, the median time between refibrillation and the next shock has decreased from 117 s to 53 s, as determined from the clinical AWC evaluation in the DEFI 2022 study.^20^

### First outcome: Global AED SAS performance during CPR

The main results of this clinical study, involving AEDs equipped with the AWC algorithm, provide evidence that the global AED SAS performance meets the AHA recommendations for arrhythmia analysis. The measured sensitivity of 94.9% (LCL90 = 93.7%) and specificity of at least 99.3% (LCL90 = 99%) exceeds the respective shockable and non-shockable performance goals (Table 1A). The one-sided 90% lower confidence limit values demonstrate reliable results.

SAS performance on clean-ECG signals is compliant with the standards,^27^, ^32^ (Table 1B) despite the duration of the AED analysis in absence of CC being substantially shortened to 5 s. It should be reminded that a shorter analysis duration is a prerequisite for fulfilling the pre-shock pauses guidelines recommendations.^5^, ^33^, ^34^

Even in presence of CC (noisy ECG signals), the AWC(Step1) meets the standards for noise-free signals in terms of VF-sensitivity and ONR-specificity (Table 1B). Only ASYS specificity (86.5%) does not achieve the required goal, which is a known weakness for low-amplitude ECGs in presence of chest-compressions.^8^, ^19^

Generally, the detection of intermediate rhythms (Table S2) is irrelevant to the first outcome and can only be reported. In this context, we report 78 cases (75%) of shock advice for the intermediate rhythm fine-VF, assessed for the global AED SAS performance. Additionally, the rhythm transitions contain signals that are of different nature, both shockable and non-shockable in unknown proportion, therefore the performance interpretation is not meaningful.

### Second outcome: Safety of AWC

Based on the previously disclosed results, one might question the necessity of conducting the AWC (Step 2) confirmation analysis. However, unnecessary shock confirmations are present and should be minimized because each confirmation implies an additional hands-off time measured at 10 s median. It has been found that only 8% of non-shockable cases led to confirmation analysis with AWC(Step2). The AWC safety analysis (Fig. 4) shows that inappropriate CoA was applied in a minority of patients (28%). Only one patient (0.35%), in asystole, had up to six ill-advised CoA, making his impact negligible over the cohort. As mentioned earlier, the incorrect classification of asystole in presence of CC is a known weakness. While minor it underscores the necessity of CoA.

### Comparison to other clinical studies

This study provides a clinical confirmation of previous *in silico* tests of the same SAS during CPR.^16^ To our knowledge, only one other clinical study has been published that reports the performance of AED rhythm analysis during chest compressions in real OHCA setting. The implemented AED algorithm is called cprINSIGHT, and its results are compared in Table 2. Similarly to AWC, the cprINSIGHT algorithm respected two-step protocol for rhythm analysis during CPR. The differences concern the shock advisory decisions taken by (Step1) analysis in presence of CC, given the cprINSIGHT ternary output for categories: shockable, non-shockable and “pause needed”. In the study of De Graaf et al.,^19^ shock was directly delivered to 81.8% of shockable rhythms and CPR was continued for 78.2% ONR and 60.3% ASYS, where performances were computed from the total number of analyses, including the “pause needed” cases, (Table 2). The AWC technology in the current clinical study provided about 11% higher proportion of VF patients to be shock-advised during CPR, as well as about 17–26% higher proportion of ONR and ASYS cases to continue with CPR. Comparison of both clinical studies after the two-step protocol (Step1 + Step2 in Table 2) shows disparities in sensitivity (91.4–95.4%) and specificity (97.5–100%), nevertheless the overall accuracy is similar and equal to 98% (1836/1874) for the AWC algorithm and 97.8% (708/724) for the cprINSIGHT algorithm.

Another issue for comparison of SAS decision policies is the number of analyses during CC that need confirmation, given that fewer CoA reduces the hands-off time. The AWC algorithm advises CoA when shockable rhythms are detected during CC, reaching CoA rate of 27.7%, Table 2. It is comparable to 29.6% CoA rate in “pause needed” cases during CPR, given by the cprINSIGHT algorithm.

CoA, provided as a second step in the AWC algorithm, takes advantage of the necessary cessation of rescuer chest compressions to deliver the shock by following voice prompts. In AEDs, several messages are provided to assure that the rescuers stepped back during the confirmation analysis without loss of time. The time between cessation of chest compressions and shock delivery is in the same order of magnitude between^19^ and the DEFI 2022 study^20^ (median 8 vs 12 s).

## Study limitations

The main limitation of this study is related to the type of analysed rhythm. Among the 285 enrolled patients, only a small number of shockable rapid-VTs (3 cases) and no non-shockable NSR were observed during CPR. Therefore, the corresponding performance results are difficult to interpret as part of the conclusion for the routine AED use during chest compressions.

Absence of results on scarce rhythms could represent a safety risk. The risk of harm is related to delivering shocks to patients presenting non-shockable rhythms, including NSR, OR and ASYS. For the primary outcome of this study (AWC global performances), NSR (16 cases) are the only rhythms under the minimum requirements for reporting performance (100 cases).^27^ The global performance and performance of the AWC(Step1) and AWC(Step2) algorithms, working separately or in sequence, have been validated on such rhythms in a previous study,^16^ showing adequacy to requirements.

Children with pediatric CPR protocol (15:2) were excluded from the study because AWC needs at least 16 s of chest compressions during CPR.

Patients with pacemakers were excluded from the analysis for two reasons. First, the SAS performance guidelines specifically state that performance goals concern non-noisy signals. Second, there was a high likelihood that these cardiac arrest rhythms would be annotated as “undefined” due to a lack of consensus between annotators.

## Conclusions

This study reports clinical experience on using AEDs with integrated algorithm for analysis of the ECG rhythm during CPR by first responders in the BLS-phase of early OHCA treatment without the need of any CPR feedback system. Up to now, it is one of the first clinical studies, providing positive evidence for the routine use of these technologies in clinical practice, as required by the International Liaison Committee on Resuscitation (ILCOR) task force guidelines.^35^ Although the presented technology embeds a confirmation analysis, this feature might not be considered a waste of time because AED messages broadcasting is run simultaneously, thus allowing an optimal reaction of the rescuer to deliver the shock.

We conclude that the global performance of the integrated AWC algorithm for rhythm analysis during CPR meets the standards with sensitivity of 94.9% and specificity of at least 99.3%. This AWC implementation provides innovative treatment of shockable rhythms by stopping CPR and deliver a shock earlier than 2 min in most VF-patients (92.9%), while most non-shockable patients (86.5– 95.2%) benefit from continuous CPR for at least 2 min.

## Registration

This study was registered under identification number NCT04691089 in ClinicalTrials.gov.

## Funding

This research received no external funding.

## CRediT authorship contribution statement

**Jean-Philippe Didon:** Writing – review & editing, Writing – original draft, Supervision, Resources, Project administration, Methodology, Conceptualization. **Irena Jekova:** Writing – review & editing, Writing – original draft, Visualization, Validation, Software, Formal analysis. **Benoît Frattini:** Writing – review & editing, Validation, Software, Resources, Methodology, Investigation, Data curation, Conceptualization. **Sarah Ménétré:** Writing – review & editing, Visualization, Validation, Software, Resources, Project administration, Methodology, Formal analysis, Data curation, Conceptualization. **Clément Derkenne:** Writing – review & editing, Visualization, Project administration, Methodology, Investigation, Data curation, Conceptualization. **Vivien Hong Tuan Ha:** Writing – review & editing, Validation, Software, Methodology, Formal analysis, Data curation, Conceptualization. **Daniel Jost:** Writing – review & editing, Supervision, Software, Resources, Methodology, Investigation, Formal analysis, Data curation, Conceptualization. **Vessela Krasteva:** Writing – review & editing, Writing – original draft, Visualization, Validation, Supervision, Software, Formal analysis.

## Declaration of competing interest

The authors declare the following financial interests/personal relationships which may be considered as potential competing interests: “The authors VK, IJ, BF, DJ, CD and VH declare no competing interests. JPD and SM are employees of SCHILLER Médical, Wissembourg, France. JPD holds the patents for the Analyze Whilst Compressing algorithm under evaluation.”.
