# Supplementary material for: Clinical performance of AED shock advisory system with integrated Analyze Whilst Compressing algorithm for analysis of the ECG rhythm during out-of-hospital cardiopulmonary resuscitation: A secondary analysis of the DEFI 2022 study
Source: Resusc Plus. 2024 Aug 5;19:100740. doi: 10.1016/j.resplu.2024.100740 (PMC11343048; doi:10.1016/j.resplu.2024.100740)
Supplement: Supplementary Data 1 [file mmc1.pdf]

Supplementary material

Clinical performance of AED shock advisory system with integrated Analyze Whilst Compressing algorithm for analysis of the ECG rhythm during out-of-hospital cardiopulmonary resuscitation: A secondary analysis of the DEFI 2022 study

Jean-Philippe Didon[1], Irena Jekova[2], Benoît Frattini[3], Sarah Ménétré[1], Clément Derkenne[3], Vivien Hong Tuan Ha[3], Daniel Jost[3], Vessela Krasteva[2]

[1] Schiller Médical SAS, 4 rue L. Pasteur, 67160 Wissembourg, France

[2] Institute of Biophysics and Biomedical Engineering, Bulgarian Academy of Sciences, Acad. G. Bonchev Str. Bl 105, 1113 Sofia, Bulgaria

[3] Paris Fire Brigade, 1 place Jules Renard, 75017 Paris, France

Table S1. Baseline patient characteristics extracted from the DEFI 2022 study *[Derkenne C, Frattini B, Menetre S, Ha VHT, Lemoine F, Beganton F, et al. Analysis During Chest Compressions in Out-Of-Hospital Cardiac Arrest Patients, A Cross/Sectional study: The DEFI 2022 Study. Resuscitation 2024, https://doi.org/10.1016/j.resuscitation.2024.110292]*

	**From 01/01/2021 to 31/01/2022**<br>**N=285**
**Age in years, median [IQR]**	60 [51–72]
**Men, n (%)**	231 (81)
**Location, n (%)**	
**Home**	171 (60)
**Public place**	97 (34)
**Other**	17 (6)
**Bystander status, n (%)**	
**Not witnessed**	10 (3.5)
**Witnessed and no CPR**	41 (14)
**Witnessed and early lay-rescuer CPR**	208 (73.2)
**BLS team witnessed**	26 (9.1)
**First monitored rhythm with shock advice, n (%)**	
**VF**	251 (88.1)
**Rapid VT**	2 (0.70)
**NSR**	0 (0)
**ONR**	3 (1.05)
**ASYS**	1 (0.35)
**Intermediate**	27 (9.47)
**Undefined**	1 (0.35)
**Call to connection of AED, min, median [IQR], [a]**	12 [10–15]
**AED connection to first defibrillation, s, median [IQR]**	19 [17–22]
**Survival at hospital admission, n (%)**	170 (59.6)
**Survival at hospital discharge, n (%)**	79 (28.0)

a: BLS team-witnessed patients were excluded.

AED: Automated External Defibrillator, CPR: cardiopulmonary resuscitation; BLS: Basic Life Support, VF: Ventricular fibrillation; VT: Ventricular tachycardia, NSR: Normal sinus rhythm; ONR: Other non-shockable rhythm; ASYS: Asystole; IQR: Interquartile range.

The DEFI 2022 study was conducted in the Greater Paris area (7 million inhabitants, 800 km2), and features a two-tier physician-manned emergency system. BLS teams managed out-of-hospital cardiac arrests according to the European Resuscitation Council guidelines. They used a DEFIGARD Touch7 AED (DGT7, SCHILLER Médical, France). Subsequently, mobile intensive care units (MICU) assisted BLS on site.

Table S2. Shock/no shock decisions during CPR for intermediate rhythms, considering the global AED shock advisory system (Standard analysis + AWC (Step 1 + Step 2)), in this clinical study.

Rhythm	Patients	Analyses	Shock advised	No shock advised	Performance Goal [27,32]
**fine VF**	34	104	78	26	NA
**slow VT**	2	2	1	1	NA
**Transition to Sh**	37	41	3	38	NA
**Transition to NSh**	18	21	0	21	NA

NA: Not Applicable; VF: Ventricular Fibrillation; VT: Ventricular Tachycardia; Sh: Shockable; NSh: Non-shockable.

"Transition to Sh" and "Transition to NSh" are non-homogeneous analysis segments in which the relative proportion of the shockable and non-shockable rhythm was not measured. This implies classifying these analysis segments as intermediate rhythms.

Table S3. Explanation of the principle of computation of the shock advisory system performance.

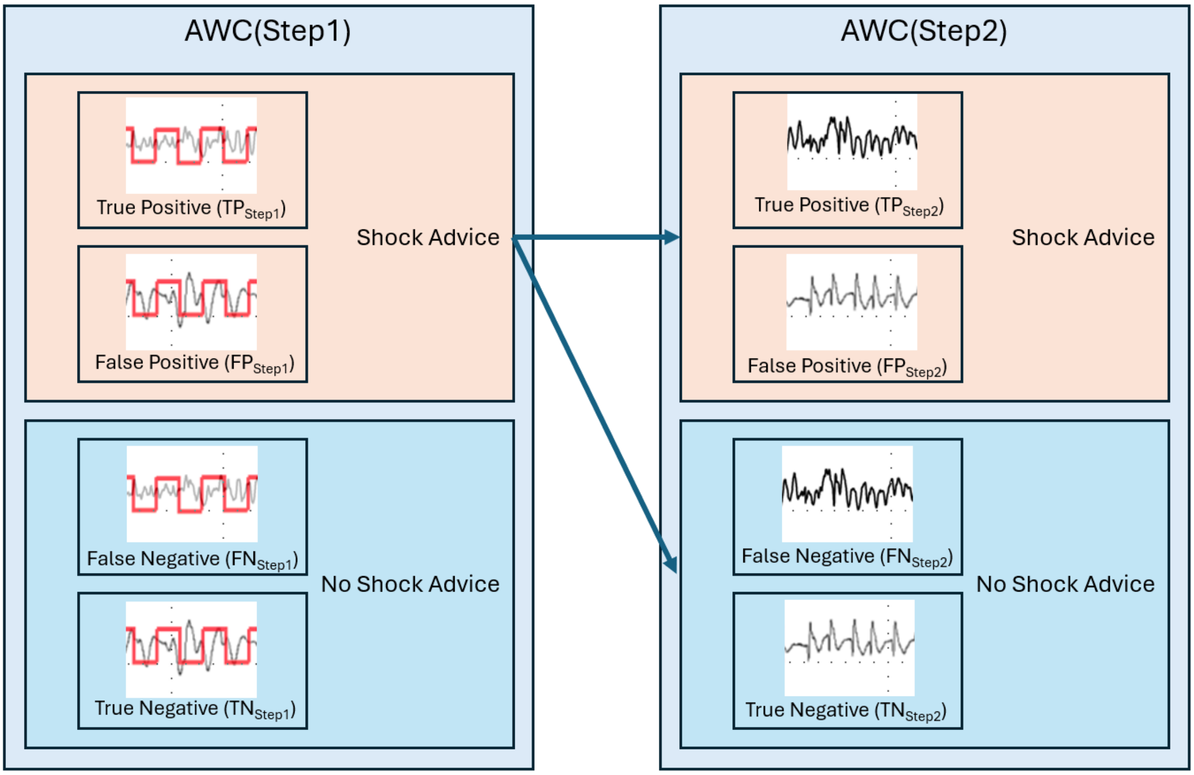

TP: True Positive; TN: True Negative; FP: False Positive; FN: False Negative.

Analyses sequence	Description	Formula
**AWC (Step 1 + Step 2)**	Combined performance of Step1 and Step2 for AWC algorithm	$Se_{Step1+2} = \frac{TP_{Step2}}{TP_{Step1} + FN_{Step1}}$ $Sp_{Step1+2} = \frac{TN_{Step1} + TN_{Step2}}{TN_{Step1} + FP_{Step1}}$
**Standard analysis + AWC (Step 1 + Step 2)**	Global performance for AWC algorithm	$Se_{Global} = \frac{TP_{Standard} + TP_{Step1+2}}{TP_{Standard} + FN_{Standard} + TP_{Step1+2} + FN_{Step1+2}} = \frac{TP_{Standard} + TP_{Step2}}{TP_{Standard} + FN_{Standard} + TP_{Step2} + FN_{Step1} + FN_{Step2}}$ $Sp_{Global} = \frac{TN_{Standard} + TN_{Step1+2}}{TN_{Standard} + FP_{Standard} + TN_{Step1+2} + FP_{Step1+2}} = \frac{TN_{Standard} + TN_{Step1} + TN_{Step2}}{TN_{Standard} + FP_{Standard} + TN_{Step1} + TN_{Step2} + FP_{Step2}}$

AWC: Analyze Whilst Compressing; Se: Sensitivity; Sp: Specificity.

Standard analysis is equivalent to AWC (Step2) without being triggered by a previous AWC (Step1)
